# Efficient extraction of proteins from formalin-fixed paraffin-embedded tissues requires higher concentration of tris(hydroxymethyl)aminomethane

**DOI:** 10.1186/1559-0275-11-4

**Published:** 2014-02-01

**Authors:** Yusuke Kawashima, Yoshio Kodera, Anil Singh, Masaomi Matsumoto, Hiroyuki Matsumoto

**Affiliations:** 1 Department of Biochemistry and Molecular Biology, University of Oklahoma Health Sciences Center, Oklahoma City, OK, USA; 2 Laboratory of Biomolecular Dynamics, Department of Physics, School of Science, Kitasato University, Kanagawa, Japan; 3 Center for Disease Proteomics, School of Science, Kitasato University, Kanagawa, Japan; 4 Department of Chemistry, University of North Carolina, Chapel Hill, NC, USA

**Keywords:** FFPE, Protein extraction, TRIS concentration, Proteomics

## Abstract

**Background:**

Numerous formaldehyde-fixed and paraffin-embedded clinical tissues have been created in the past decades and stored in pathological depositories at hospitals as well as in clinical laboratories worldwide. In addition to the archived tissues, formaldehyde-fixation is also mandatory for preparing proteomics samples from diseased patients or animal models in order to inactivate contagious agents. Protein extraction from formaldehyde-fixed tissues is hampered by the Schiff base formation between the amino groups of proteins and formaldehyde. Although achievement of the highest extraction efficiency of proteins from the formaldehyde-fixed tissues is essential for obtaining maximum proteomics information, no attention has been paid to the concentration dependence of tris(hydroxymethyl)aminomethane on the extraction efficacy. We suspected that the concentration of tris(hydroxymethyl)aminomethane affects the protein extraction efficiency because of its property as a primary amine that reverses the Schiff base formation between the primary amines of proteins and formaldehyde. Thus we pursued optimization of the component and protocol of protein extraction buffer to achieve better extraction efficiency of proteins from formaldehyde-fixed and paraffin-embedded tissues.

**Results:**

In order to simulate protein extraction from diseased tissues we made formaldehyde-fixed and paraffin-embedded samples from mouse liver slices and investigated the protein extraction efficiency and speed by changing the concentration of the protein extraction buffer component tris(hydroxymethyl)aminomethane under various extraction conditions. We find, as expected, that tris(hydroxymethyl)aminomethane significantly affects the performance of protein extraction from the formaldehyde-fixed and paraffin-embedded samples both in the extraction yield and in the extraction speed.

**Conclusions:**

We recommend the concentration of tris(hydroxymethyl)aminomethane in protein extraction buffer to be higher than 300 mM when extraction is conducted for 90 min at 90°C to achieve the most efficient protein extraction in a shorter time. The information will be essential for performing the most efficient protein extraction from formaldehyde-fixed and paraffin-embedded tissue samples for proteomics analysis.

## Background

Formaldehyde fixation and paraffin embedding (FFPE) is a routine procedure for histopathological diagnosis of disease. FFPE tissues are highly stable, and can be stored at room temperature indefinitely. Therefore, large repositories of healthy as well as pathological FFPE tissues have been generated and stored worldwide. These FFPE samples are associated with clinical information concerning diagnosis, treatment, and outcome of the patient, and mainly serve as specimens for pathological investigation. Meanwhile, proteins are generally preserved for a long time even at room temperature; for example, we had demonstrated that two-dimensional gels that have been archived and stored over two decades can still serve as a source for proteomics analysis [1]. In a similar context, we can regard FFPE tissues as a source for invaluable materials for proteomics analysis for the molecular characterization of disease processes aimed at improving diagnosis, prognosis, and therapy [2]. One disadvantage of formalin fixation is significant cross-linking between proteins and other molecules in the tissue through Schiff base formation [3], hampering molecular investigation of these samples. The chemical cross-linking inactivates biochemical and pathological processes and, therefore, is an essential step for the investigation of organ specimens from an animal or human body that suffered from lethal pathogens. For example, complete inactivation of dangerous pathogens such as *Bacillus anthracis* infection [4] or Ebola virus infection [5] is essentially important for the biosafety issue. In any case, for an effective proteomics analysis of necropsy or biopsy specimens, effective protein extraction from FFPE tissues is an absolute requirement [6].

Based on the principle of heat-induced antigen retrieval technique, Ikeda *et al*. first described protein extraction from FFPE tissue in 1998 [7]. Recently, different research groups have reported success by protein extraction from FFPE tissue coupled with gel-based proteome analysis approaches, such as 2-D PAGE [8], 2-D DIGE [9], and 1-D PAGE followed by LC–MS/MS (GeLC–MS/MS) [10]. To overcome proteomics of wider range, several groups have improved efficiency of protein extraction from FFPE tissues. Especially, components of protein extraction buffer (PEB) are well investigated, such as the pH of buffer, the concentrations of SDS in buffer with or without DTT [11-17]. Many reported PEBs used tris(hydroxymethyl)aminomethane (TRIS) as a buffering agent to stabilize pH. However, in all the published reports of FFPE tissue proteomics, the concentration effect of TRIS on protein extraction in PEB has never been investigated probably because it seems unlikely that the presence of TRIS in PEB would assist protein extraction in other context than for stabilizing the solution pH. From this reason the TRIS concentration in PEBs have been kept relatively low in the 10 mM range. The use of moderate concentration of TRIS is also the case in all of the works presented in the most recent special issue dedicated to FFPE proteomics analysis featured in Proteomics-Clinical Applications; for example, [18,19]. In this paper, we evaluated the TRIS concentration effect on protein extraction efficiency from FFPE tissues and would like to recommend minimum requirement for the TRIS concentration in order to achieve optimized FFPE proteomics analysis.

## Results

**Figure 1:**
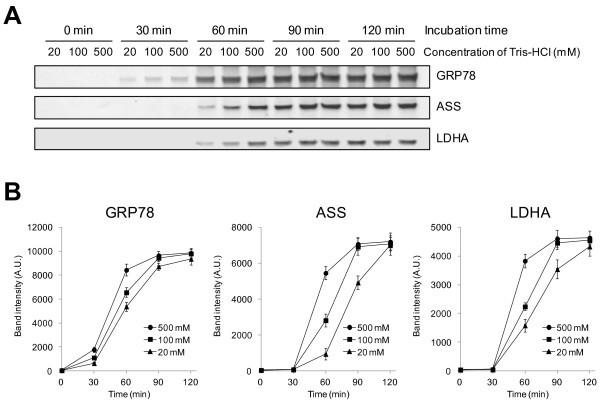
**TRIS effect of on protein extraction efficacy from FFPE liver tissues probed by Western blots.** FFPE liver was deparaffinized, and homogenized in 20, 100 and 500 mM TRIS–HCl, pH 8.0, respectively, all containing 2% SDS. Subsequently, proteins were extracted from tissue homogenate by incubating at 90°C for 0, 30, 60, 90 and 120 min, respectively. **(A)** Western blotting against Grp78, Ass and Ldha of proteins extracted from FFPE liver tissue. **(B)** The densitometric comparison of the band intensities in **(A)**. The mean and SD values of band intensities for three independent experimental triplicates (n = 3) are shown.

For FFPE tissues we used liver tissues of male C57BL/6 mice that were fixed by 10% formaldehyde and embedded in paraffin. First, we examined dependence of protein extraction efficiency from FFPE mouse liver on 20 mM, 100 mM and 500 mM TRIS, respectively, in PEB. The incubation time during the heat-treatment was also varied; 0, 30, 60, 90 and 120 min. After the treatment, the protein extraction efficiency was evaluated by band intensities of Grp78, Ass and Ldha observed in Western blotting (Figure 1). The band intensities of proteins drastically increased with prolonged incubation time of the heat-treatment. Without heat-treatment, all PEBs containing various concentrations of TRIS failed to extract proteins effectively (0 min in Figure 1A). With the heat treatment for 30 min, band intensities of Grp78 were increased with higher concentration of TRIS, whereas the bands of Ass and Ldha were not detected. With heat treatment for 60 min, the band intensities of Grp78, Ass and Ldha increased with higher concentration of TRIS. With the heat treatment for 90 min, the band intensities of three proteins extracted by 100 mM and 500 mM TRIS were similar, whereas those of 20 mM TRIS PEB were distinguishably weaker. Thus the protein extraction at 100 mM and 500 mM TRIS-containing PEB maxed out during the 90 ~ 120 min of incubation time. With the heat treatment for 120 min, the proteins extraction by 20 mM TRIS-containing PEB also reached at the limit of protein extraction efficiency. Therefore, we conclude that the protein extraction from formalin fixed tissue was enhanced and accelerated with increasing concentration of TRIS in the PEB.

**Figure 2:**
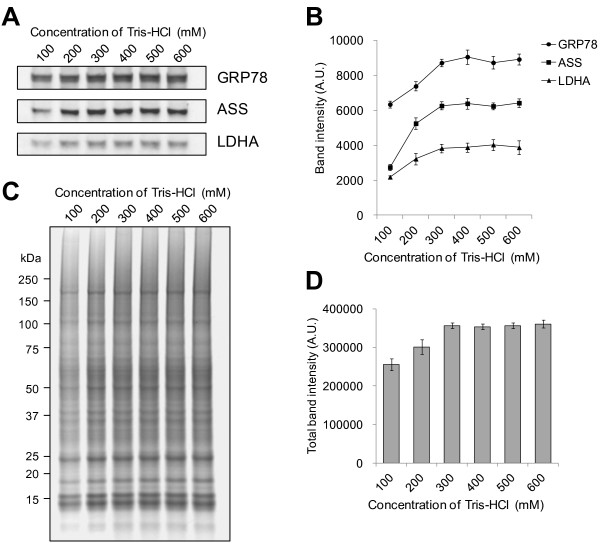
**TRIS concentration-dependence of the protein extraction efficacy as monitored by Grp78, Ass, and Ldha holds to other proteins.** FFPE liver was deparaffinized, and extracted by 100, 200, 300, 400, 500 and 600 mM TRIS–HCl, pH 8.0, respectively, all containing 2% SDS at 90°C for 60 min. **(A)** Western blotting against Grp78, Ass, and Ldha in FFPE mouse liver extracted by PEBs containing 100 ~ 600 mM TRIS. **(B)** Densitometric comparison of the band intensities in **(A). (C)** SDS-PAGE profiling of proteins in FFPE mouse liver tissue extracted by PEBs containing 100-600 mM TRIS. **(D)** Densitometric comparison of the total band intensities in **(C)**. The mean and SD values of band intensities for three independent experimental triplicates (n = 3) are shown.

With the survey data on the extraction efficacy and speed by TRIS as shown in Figure 1, we next determined the necessary and sufficient concentration of TRIS component in the PEB for the maximum yield in protein extraction from FFPE tissues. Figure 2 shows the comparison of extraction efficiency of proteins extracted by 100, 200, 300, 400, 500 and 600 mM TRIS in PEB at 90°C for 60 min. This condition of heat-treatment was chosen because Grp78, Ass and Ldha observed by Western blotting and extraction efficiency of those proteins didn’t reach at the maximum. The band intensities of Grp78, Ass and Ldha detected by Western blotting were increased with concentration of TRIS from 100 to 300 mM, and saturated beyond 300 ~ 600 mM (Figure 2A and B). CBB-stained profiles of SDS-PAGE are consistent with the TRIS-dependent protein extraction efficiency as exemplified by the Western blot results of Grp78, Ass and Ldha (Figure 2C and D). Since the SDS-PAGE profile is likely to represent most of the soluble protein species including Grp78, Ass, and Ldha as well as some membrane-associated proteins and membrane proteins although in minor quantities, we interpret the data that the efficient extraction conditions we establish here could be applicable to most protein species. Thus, the results indicate that 300 mM TRIS in PEB is at least necessary and very likely sufficient as well to extract proteins quickly and effectively from the FFPE samples.

**Scheme 1:**
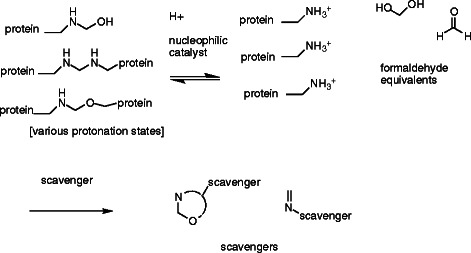
TRIS acting as a formaldehyde scavenger, producing Schiff base, cyclic hemiaminal, and cyclic acetal adducts.

We note that the TRIS concentration in PEB has been used at the several tens of mM probably because the authors are simply following a conventional recipe for protein extraction practice. In this work we showed significant concentration dependence of TRIS in PEB upon the protein extraction efficiency. The mechanism of this extraction enhancement appears to originate in the chemical property of formaldehyde to modify and crosslink proteins via Schiff base formation [3] that is essentially reversible. There are at least two mechanisms that could be involved in the TRIS-catalyzed enhancement of protein extraction from FFPE tissues. First is caused by the TRIS molecule acting as a scavenger to remove released formaldehyde as shown below (Scheme 1).

In this scheme, TRIS is acting as a formaldehyde scavenger, producing Schiff base, cyclic hemiaminal, and cyclic acetal adducts. The reactions of formaldehyde and amines are highly reversible under normal conditions, so removing formaldehyde from equilibrium will greatly favor de-crosslinking.

**Scheme 2:**
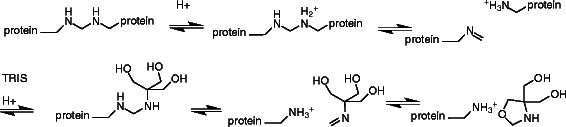
TRIS directly involved in breaking down the crosslink.

It is also possible that TRIS is directly involved in breaking down the crosslink, as a kind of transamination catalyst as illustrated in the following scheme (Scheme 2).

In this capacity, the increased effective molarity of the three hydroxyl groups of TRIS may act to trap the Schiff base intermediate before it can re-crosslink. In order for this to work, there would have to be a high concentration of TRIS, which is consistent with our findings. These two mechanisms are not mutually exclusive. In any case, in order to achieve the maximum effectiveness of FFPE proteomics analysis, it is essential to explore and optimize the de-crosslinking reaction of formaldehyde-modified amines. In the same special issue dedicated to FFPE proteomics a powerful shotgun analysis achieved an impressive depth of 10,000 proteins from FFPE tissues [18]. In a shotgun proteomics analysis, proteins are digested in diverged physical states ranging from a mono-dispersed state to multiply oligomeric configurations both in non-covalent and covalent bonding. Since modern mass spectrometry is sensitive and accurate to achieve reliable false discovery rate (1% in the case of reference [18]) with a smaller number of digested peptides, complete de-crosslinking is unnecessary for protein identification. In contrast, under our optimized condition we achieved extraction of mono-dispersed or nearly mono-dispersed protein molecules as exemplified with the proteins probed with antibodies. In any case, an effective and standardized solubilization from FFPE samples would be required to maximize proteomics information from FFPE tissue samples [5].

## Conclusions

We showed that the concentration of TRIS in PEB drastically influenced protein extraction from the FFPE tissue samples. Proteins in the FFPE tissue were quickly and effectively extracted dependent on the increasing concentration of TRIS, and the extraction efficiency saturated at TRIS concentration higher than 300 mM. Therefore, in contrast to the conventional concentrations of TRIS in the several tens of mM range, we recommend concentration of TRIS in PEB at higher than 300 mM for a better and quicker extractability of proteins. This information is crucial for future proteome studies of wide variety of FFPE tissues aimed at discovery and development of new diagnostic markers and therapeutic targets.

## Materials and methods

For all experiments, we used liver tissues obtained from male C57BL/6 mice. The animal protocol was approved from the Institutional Animal Care and Use Committee of the University of Oklahoma Health Science Center. Tissues were fixed in 4% buffered formalin for 48 h, dehydrated with a graded series of ethanol before being embedded in paraffin. FFPE tissue sections were transferred to 1.5 ml polypropylene microcentrifuge tubes and deparaffinized by incubation at room temperature in xylene for 10 min. After incubation, the tissue was pelleted at 16,000 × g for 3 min, and the incubation/centrifugation steps were repeated twice. The deparaffinized tissue pellets were then rehydrated with a graded series of ethanol, briefly air-dried in a fume hood, and weighed. Then, the tissue pellets were homogenized using a PowerGen 125 Tissue Homogenizer (Thermo Fisher Scientific, Waltham, MA) in 100 volumes of PEB (20-600 mM Tris–HCl pH 8.0 and 2% SDS). Samples were incubated at 90°C for 0-120 min. The extracts were centrifuged for 20 min at 16,000 × g at 4°C, and 4 μL of each supernatant was subjected to SDS-PAGE (NuPAGE 4-12% Bis-Tris gel; Invitrogen, Carlsbad, CA) according to the manufacturer’s instruction. The gels were stained by CBB R-250, and destained in 25% 2-propanol containing 10% acetic acid. For Western blotting, electrophoresed proteins were transferred onto a PVDF membrane (Immobilon-FL, pore size: 0.45 μm; EMD Millipore, Billerica, MA) at 10 V for overnight in a tank transfer apparatus (Bio-Rad Laboratories, Hercules, CA). The membranes were blocked in Odyssey Blocking Buffer (Li-Cor Biosciences, Lincoln, NE) for 1 h at room temperature with constant shaking. Primary antibodies diluted 1:1000 in Odyssey Blocking Buffer were incubated with membranes at room temperature for 2 h. The membranes were washed three times with TBS plus 0.1% Tween-20, and were incubated for 1 h with the specific secondary anti-goat IgG antibody conjugated to IRDye 800CW (Li-Cor Biosciences). Secondary antibodies were used at dilutions 1:10000 in Odyssey Blocking Buffer. After three washes with TBS plus 0.1% Tween-20, the Proteins were visualized using fluorescent-labeled secondary antibodies and quantified by Odyssey Infrared Imaging System (Li-Cor Biosciences). Primary antibodies used in this work were polyclonal goat anti-78 kDa glucose-regulated protein (Grp78) antibody, polyclonal goat anti-argininosuccinate synthase (Ass) antibody, and polyclonal goat anti-lactate dehydrogenase A (Ldha) antibody (Santa Cruz Biotechnology, Santa Cruz, CA). The quantitation of bands of SDS-PAGE and Western blotting were estimated by densitometric analysis using ImageJ software (National Institutes of Health).

## Abbreviations

FFPE: Formaldehyde-fixed paraffin-embedded; PEB: Protein extraction buffer; TRIS: Tris(hydroxymethyl)aminomethane; Grp78: 78 kDa glucose-regulated protein; Ass: Argininosuccinate synthase; Ldha: Lactate dehydrogenase A.

## Competing interests

The authors declare that they have no competing interests.

## Authors’ contributions

YK (Kawashima) has been involved in planning of study, collection and evaluating data and manuscript writing. YK (Kodera) has been involved in evaluating and analyzing the data and writing the manuscript. AS has been responsible for collection of materials data. MM is involved in interpreting the mechanisms of TRIS-enhanced protein solubilization. HM has been involved in the planning of the study, data evaluation and writing the manuscript. All authors read and approved the final manuscript.
